# Addison crisis related psychosis

**DOI:** 10.1192/j.eurpsy.2022.1205

**Published:** 2022-09-01

**Authors:** A. Papanastasiou, A. Roubi, L. Tsitrouli, A. Antoniou, G. Vouraki, E.T. Tsapardoni, V. Drakuli, S.M. Papageorgiou, A. Pahi

**Affiliations:** SOTIRIA GENERAL UNIVERSITY HOSPITAL, Psychiatry, ATHENS, Greece

**Keywords:** delusion, Addison, cortizole, Psychosis

## Abstract

**Introduction:**

Addison’s disease (AD) is a rare disorder of the adrenal glands which causes deficiency of cortisol and aldosterone. It presents with a variety of symptoms, including neuropsychiatric manifestations. We discuss the case of a patient who exhibited psychotic symptoms in clear consciousness and no other clinical sign of AD.

**Objectives:**

To investigate the association between AD and neuropsychiatric symptoms; to make clinicians aware of psychotic manifestations of AD as first presentation.

**Methods:**

Case Presentation of a patient with psychosis and AD. A review of the literature was conducted in PubMed using the following keywords: Addison’s disease, Addison crisis, psychosis, psychotic, neuropsychiatric

**Results:**

A 32-year-old alert male patient presented with delusions of persecution, auditory hallucinations and mild psychomotor agitation after a stressful life event. Lab tests showed hyponatremia (132 mEq/L). Patient exhibited rapid clouding of consciousness after admission and further lab results showed low levels of cortisol. He was therefore started treatment with high doses of hydrocortisone with good response. A close association between AD and psychiatric manifestations was indicated by the literature review, especially in males and those with thyroid dysfunction comorbidity. These include a wide range of symptoms, such as apathy, catatonia, anxiety, depression, lethargy, delirium, cognitive disorder, irritability, behavioural disorders, agitation, delusions, hallucinations, and rarely psychotic symptoms in clear consciousness. The aetiopathogenetic mechanism involves electrolyte disturbances, cortisole deficiency and increase in endogenous endorphines

**Conclusions:**

Clinicians should be alert of the manifestation of AD with psychiatric symptoms ;patients with AD should be informed of the risk for Addison crisis after stress.

**Disclosure:**

No significant relationships.

